# Longitudinal Clinical and Microscopic Findings After Dequalinium Chloride Treatment in Vulvovaginal Infections: A Retrospective Real-World Study from Mexican Gynecological Practice

**DOI:** 10.3390/jcm15156115

**Published:** 2026-08-06

**Authors:** Gerardo Casanova-Román, Carolina Navarro-Venegas, Verónica Ávalos-López, Cinthya Verver-Moreno, Henry Velazquez-Soto, Nataly Arellano-Contreras, Isabel Garza Ramos Raya, Israel Casanova-Méndez, on behalf of the AMEGINE Clinical Research Group

**Affiliations:** 1 Cell Biology and Medical Histology, Saint Luke School of Medicine, Mexico City 11000, Mexico; dr_casanova_ets@hotmail.com; 2 Gynecology and Obstetrics, Angeles Hospital, Mexico City 11800, Mexico; 3 Department of Immunology and Research Unit, Institute of Ophthalmology “Conde de Valenciana Foundation”, Mexico City 06800, Mexico; 4 Department of Non-Ophthalmological Emergencies and Research Unit, Institute of Ophthalmology “Conde de Valenciana Foundation”, Mexico City 06800, Mexico

**Keywords:** dequalinium chloride, bacterial vaginosis, vulvovaginal candidiasis, mixed vaginal infection, gynecological infection

## Abstract

**Background**: Vulvovaginal candidiasis, bacterial vaginosis, and mixed vaginal infection are among the most common infectious conditions encountered in gynecological practice. Although intravaginal dequalinium chloride exhibits broad antimicrobial activity, real-world longitudinal data describing treatment-associated clinical and microscopic changes remain limited, particularly in Latin American populations. **Objective**: We aimed to characterize the short-term longitudinal evolution of clinical manifestations, vaginal pH, and fresh wet-mount microscopic findings in women with vulvovaginal candidiasis, bacterial vaginosis, and mixed vaginal infection treated with intravaginal dequalinium chloride during routine outpatient gynecological care. **Methods**: This retrospective longitudinal study included non-pregnant women with vulvovaginal candidiasis, bacterial vaginosis, or mixed vaginal infection who received intravaginal dequalinium chloride as the sole anti-infective therapy. Patients were evaluated at baseline, treatment completion (Day 7), and post-treatment follow-up (Day 14). Longitudinal assessment included patient-reported symptoms, physician-assessed clinical findings, vaginal pH, and fresh wet-mount microscopic examination. To ensure consistent evaluation across study visits, predefined protocol-based standardized assessment criteria were applied uniformly to all patients. Repeated-measures non-parametric analyses were performed to compare longitudinal changes over time. **Results**: Sixty-nine women were included, comprising those with vulvovaginal candidiasis (n = 24), bacterial vaginosis (n = 31), and mixed vaginal infection (n = 14). Significant longitudinal improvement was observed in clinical manifestations, vaginal pH, and fresh microscopic findings across all diagnostic groups (all *p* < 0.0001). Median composite assessment scores decreased from 8 (IQR 6–10) at baseline to 3 (IQR 1–5) at treatment completion and to 1 (IQR 0–3) at post-treatment follow-up. Improvements in vaginal pH were accompanied by reductions in inflammatory and infection-associated microscopic findings and by increased predominance of Döderlein bacilli during follow-up. Intravaginal dequalinium chloride was generally well tolerated, with only mild transient adverse events documented. **Conclusions:** In this retrospective longitudinal study, intravaginal dequalinium chloride treatment was associated with short-term changes in clinical manifestations, vaginal pH, and fresh microscopic findings among women with vulvovaginal candidiasis, bacterial vaginosis, and mixed vaginal infection. These findings describe short-term treatment-associated changes observed in routine clinical practice but do not establish comparative efficacy, causality, or microbiological cure. Prospective controlled studies incorporating standardized microbiological endpoints and longer follow-up are warranted to confirm these observations.

## 1. Introduction

Infectious vulvovaginal disorders remain among the most common reasons for gynecological consultation in women of reproductive age and continue to pose substantial diagnostic and therapeutic challenges due to overlapping clinical manifestations, recurrent disease patterns, heterogeneous etiological profiles, and the limited diagnostic accuracy of routine clinical assessment [1]. Bacterial vaginosis, vulvovaginal candidiasis, and mixed vaginal infections frequently present with overlapping clinical manifestations, including abnormal vaginal discharge, pruritus, burning sensation, irritation, malodor, and inflammatory mucosal changes, making etiological differentiation challenging in routine clinical practice. Furthermore, mixed infectious presentations and alterations in vaginal microbial homeostasis are increasingly recognized in real-world gynecological settings, where clinical symptoms do not always correlate with a single microbiological entity and may reflect complex interactions between multiple pathogens and vaginal dysbiosis [2]. Consequently, current clinical guidelines emphasize that the diagnosis of infectious vaginitis should not rely solely on symptom assessment, as considerable overlap exists among common vaginal disorders; instead, the diagnostic approach should ideally integrate vaginal pH measurement and fresh microscopic examination whenever available, improving diagnostic accuracy, facilitating etiological differentiation, and supporting appropriate therapeutic decision-making [3,4].

Bacterial vaginosis is characterized by disruption of the *Lactobacillus*-dominant vaginal microbiota and its replacement by diverse anaerobic bacterial communities, leading to alterations in vaginal homeostasis that are clinically reflected by elevated vaginal pH, abnormal vaginal discharge, amine odor, and the presence of clue cells on microscopic examination [5]. This dysbiotic condition is clinically relevant not only because of its symptomatic burden and high recurrence rates, but also because it has been associated with an increased risk of sexually transmitted infections, pelvic inflammatory disease, adverse reproductive outcomes, and pregnancy-related complications, highlighting its broader implications for women’s reproductive health [3,6]. Vulvovaginal candidiasis, in contrast, is primarily associated with the overgrowth of *Candida* species, most commonly Candida albicans, and typically presents with vulvovaginal pruritus, burning sensation, erythema, and abnormal vaginal discharge. Nevertheless, these clinical manifestations frequently overlap with those observed in other vulvovaginal infectious disorders, particularly in mixed infections and routine outpatient gynecological practice, limiting the diagnostic accuracy of symptom-based assessment alone [7]. Consequently, etiological differentiation based solely on clinical symptoms may be challenging because of the substantial overlap among common vulvovaginal infectious disorders. This limitation reinforces the importance of complementary diagnostic evaluation, including vaginal pH measurement and, when available, fresh microscopic examination, to improve diagnostic accuracy and guide appropriate therapeutic decision-making [1,3].

The vaginal microbiological microenvironment plays a central role in the pathophysiology of vulvovaginal infectious disorders. In healthy reproductive-aged women, the vaginal ecosystem is typically dominated by *Lactobacillus* species, which contribute to the maintenance of vaginal homeostasis through lactic acid production, preservation of an acidic vaginal pH, competitive exclusion of pathogenic microorganisms, and modulation of local mucosal immune responses [8].

Disruption of this physiological balance may promote bacterial dysbiosis, fungal proliferation, inflammatory mucosal alterations, or simultaneous polymicrobial involvement [8,9]. This biological and clinical complexity partially explains why isolated symptom assessment may be insufficient to reflect disease activity or therapeutic response in routine gynecological practice. Consequently, an integrated evaluation that incorporates symptoms, physician-assessed clinical findings, vaginal pH alterations, and fresh microscopic examination may provide a more clinically meaningful interpretation of longitudinal treatment-associated changes than symptom evaluation alone.

Dequalinium chloride is a locally administered quaternary ammonium compound with broad antimicrobial activity against bacterial and fungal pathogens commonly associated with vulvovaginal infectious disorders [10]. Unlike systemic antimicrobial therapies, intravaginal dequalinium chloride acts directly at the site of infection and inflammatory alteration, providing high local therapeutic exposure with limited systemic absorption. These characteristics may be particularly relevant in routine gynecological practice, where overlapping infectious presentations, mixed microbiological findings, and heterogeneous clinical manifestations frequently complicate pathogen-oriented therapeutic selection [11]. Previous randomized clinical trials have demonstrated that intravaginal dequalinium chloride is effective in bacterial vaginosis and may achieve clinical outcomes comparable to those for standard antimicrobial therapies while maintaining favorable tolerability and local safety profiles [10,12]. Additionally, prior reviews have highlighted its potential utility as a broad-spectrum local therapeutic alternative in vulvovaginal infectious conditions involving bacterial dysbiosis, fungal overgrowth, or combined infectious presentations [13].

A particularly relevant characteristic of dequalinium chloride is its potential applicability in routine gynecological scenarios where infectious presentations are not always microbiologically isolated and where patients frequently demonstrate overlapping bacterial, fungal, inflammatory, or mixed clinical–microscopic findings. In daily outpatient practice, therapeutic decision-making often occurs before complete microbiological characterization is available, increasing the clinical importance of local therapeutic strategies capable of addressing heterogeneous vulvovaginal infectious profiles [11].

Previous reviews evaluating intravaginal dequalinium chloride have described favorable tolerability, broad local antimicrobial activity, and practical utility in vaginal infections of different etiologies, supporting its incorporation into routine gynecological management algorithms [11,14].

Despite the available literature, most published studies evaluating dequalinium chloride have been conducted in European or non-Latin American populations, whereas real-world longitudinal data from Mexican gynecological practice remain comparatively limited [14]. Additionally, many previous investigations have focused primarily on isolated diagnostic entities or binary therapeutic outcomes, even though routine gynecological practice frequently requires multidimensional interpretation of clinical symptoms, vaginal physiological alterations, and direct microscopic findings across heterogeneous infectious presentations [15,16].

In this context, longitudinal integrated evaluation incorporating physician-assessed clinical manifestations, vaginal pH, and fresh microscopic examination may provide a more clinically meaningful characterization of treatment-associated changes than isolated symptom resolution alone. The present study, therefore, aimed to evaluate the longitudinal clinical, physiological, and fresh microscopic response associated with intravaginal dequalinium chloride therapy in non-pregnant Mexican women with bacterial vaginosis, vulvovaginal candidiasis, or mixed vaginal infection during routine gynecological practice.

## 2. Materials and Methods

### 2.1. The Study Design and Setting

A retrospective longitudinal observational study was conducted to evaluate treatment-associated changes in clinical manifestations, vaginal pH, and fresh wet-mount microscopic findings among women with vulvovaginal candidiasis, bacterial vaginosis, or mixed vaginal infection treated with intravaginal dequalinium chloride during routine outpatient gynecological care.

The study was based on anonymized clinical information contributed by physicians participating in the AMEGINE Clinical Research Group. Data were retrospectively extracted from medical records generated during routine gynecological consultations conducted between November 2024 and April 2025.

Longitudinal evaluation included three predefined study visits: baseline before treatment initiation (Visit 1), treatment completion (Visit 2; Day 7), and post-treatment follow-up seven days after treatment completion (Visit 3; Day 14). This schedule allowed for assessment of both the immediate treatment response and the short-term evolution of clinical manifestations, vaginal physiological parameters, and fresh microscopic findings.

To ensure consistency across participating physicians, all clinical, physiological, and microscopic evaluations were performed according to predefined protocol-based assessment criteria established before data extraction and statistical analysis.

### 2.2. Study Population

The study population consisted of medical records of non-pregnant women of reproductive age who attended routine outpatient gynecological consultations and were diagnosed with bacterial vaginosis, vulvovaginal candidiasis, or mixed vaginal infection during standard clinical practice.

Eligible cases were identified through retrospective review of medical records. To be included in the final analytical cohort, records were required to document a complete treatment episode managed exclusively with intravaginal dequalinium chloride and to contain complete longitudinal information from all predefined study visits, including clinical manifestations, gynecological examination findings, vaginal pH measurements, and fresh wet-mount microscopic examinations.

Diagnostic classification had been established prospectively by the treating gynecologist during routine outpatient care, before initiation of this retrospective study, and independently of the present research protocol. No diagnostic procedures were performed for research purposes, and no diagnostic reclassification was undertaken during the retrospective review. Medical records were reviewed solely to confirm eligibility and to verify the consistency of the treating gynecologist’s original diagnosis with the documented clinical, vaginal pH, and fresh wet-mount microscopic findings according to the predefined study criteria. Records lacking complete baseline fresh wet-mount microscopy were excluded because the available documentation was insufficient to verify this diagnostic concordance.

Records were excluded if patients were pregnant; had received concomitant antimicrobial, antifungal, probiotic, corticosteroid, or other therapies that could influence treatment response; had concomitant gynecological conditions that could interfere with outcome assessment; or lacked sufficient clinical, physiological, microscopic, or follow-up information for longitudinal evaluation.

**Figure 1:**
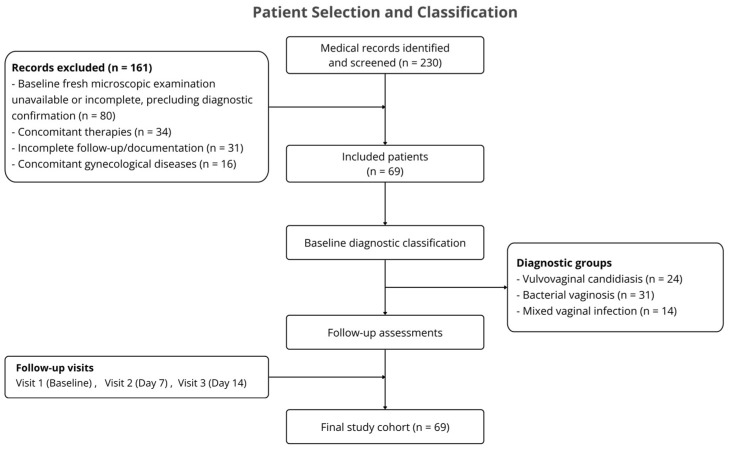
Study flow diagram. Selection of medical records included in the final analytical cohort. Of 230 medical records screened, 161 were excluded because of unavailable or incomplete baseline fresh wet-mount microscopy, concomitant therapies, incomplete follow-up or documentation, or concomitant gynecological diseases. The final analytical cohort consisted of 69 treatment episodes with physician-established diagnoses of vulvovaginal candidiasis, bacterial vaginosis, or mixed vaginal infection, evaluated longitudinally at baseline, treatment completion (Day 7), and post-treatment follow-up (Day 14).

A total of 230 medical records were screened for eligibility. Following application of the predefined inclusion and exclusion criteria, 161 records were excluded (Figure 1), the principal reason being the absence or incompleteness of the baseline fresh wet-mount microscopic examination (n = 80), which precluded retrospective verification of concordance between the original diagnosis established by the treating gynecologist and the documented clinical and microscopic findings. Additional reasons included concomitant therapies (n = 34), incomplete longitudinal follow-up or insufficient clinical documentation (n = 31), and concomitant gynecological conditions that could interfere with interpretation of treatment-associated outcomes (n = 16). The remaining 69 records fulfilled all eligibility criteria and constituted the final longitudinal analytical cohort.

### 2.3. Clinical and Gynecological Assessment

Patients underwent a standardized gynecological evaluation at each study visit, including baseline before treatment initiation (Visit 1), treatment completion (Visit 2; Day 7), and post-treatment follow-up (Visit 3; Day 14). Evaluations were performed by the treating gynecologists according to predefined protocol-based criteria routinely used during the assessment of vulvovaginal infectious disorders. These timepoints were established to include the 6-day approved manufacturer’s prescribing information.

Clinical assessment included documentation of symptoms and gynecological findings commonly associated with vulvovaginal infection, including vaginal discharge characteristics, vulvovaginal pruritus, burning or irritation, malodor, and other clinically relevant findings identified during routine examination.

To facilitate longitudinal comparison across study visits, predefined clinical variables were recorded as present or absent according to standardized criteria established before data extraction and statistical analysis. For the purpose of longitudinal assessment, the presence or absence of each clinical finding contributed 1 or 0 points, respectively, to the disease-specific clinical assessment framework.

Because characteristic clinical manifestations differ among vulvovaginal candidiasis, bacterial vaginosis, and mixed vaginal infection, disease-specific clinical assessment frameworks were applied according to the diagnostic classification of each case. The clinical variables incorporated into each disease-specific assessment framework are summarized in Table 1.

### 2.4. Vaginal pH Assessment

Vaginal pH was measured by the treating gynecologist during each routine outpatient visit as part of the standard clinical evaluation of vulvovaginal infection. Measurements were obtained using Merck^®^ MQuant™ colorimetric pH indicator strips (Merck KGaA, Darmstadt, Germany), which were applied directly to vaginal secretions collected during gynecological examination, and vaginal pH values were determined by visual comparison with the manufacturer’s standardized color reference scale.

No additional pH measurements were performed for research purposes. All pH values analyzed in the present study were obtained during routine clinical care before initiation of this retrospective investigation and were retrospectively extracted from the corresponding medical records.

For the exploratory composite clinical–microscopic assessment framework, a novel, in-house, non-validated assessment tool was developed: vaginal pH was classified as normal or altered according to the predefined disease-specific criteria for each diagnostic category with an altered or normal pH contributing 1 point or 0 points, respectively, to the disease-specific composite score. Longitudinal changes in vaginal pH were evaluated across the three predefined study visits.

### 2.5. Fresh Microscopic Examination

Fresh wet-mount microscopic examination, with and without potassium hydroxide, was performed by the treating gynecologist during each routine outpatient visit as part of the standard clinical evaluation of vulvovaginal infection. Vaginal secretions obtained during gynecological examination were immediately analyzed using direct light microscopy according to routine fresh wet-mount preparation techniques employed in outpatient gynecological practice.

Microscopic assessment included the evaluation of characteristic findings associated with vulvovaginal infectious disorders, including clue cells, leukocytes, Döderlein bacilli, yeasts, pseudohyphae, and other relevant inflammatory or microbiological findings. Representative microscopic findings corresponding to vulvovaginal candidiasis, bacterial vaginosis, and mixed vaginal infection are presented in Supplementary Figure S1.

Because preservation or restoration of lactobacillary predominance represents an important indicator of vaginal microbiological homeostasis, particular attention was given to the assessment of Döderlein bacilli during follow-up.

For longitudinal analysis, microscopic findings documented in the medical records were graded semi-quantitatively according to their recorded abundance using a predefined four-level scale (0–3), where 0 indicated absence, 1 scarce findings, 2 moderate findings, and 3 abundant findings. Particular emphasis was placed on clue cells, yeasts, pseudohyphae, inflammatory cellularity, and alterations in normal vaginal flora. The semi-quantitative grading system is summarized in Table 2.

### 2.6. Exploratory Composite Clinical–Microscopic Assessment Framework

To facilitate standardized longitudinal assessment across study visits, a disease-specific exploratory composite clinical–microscopic assessment framework was developed *a priori* before data extraction and statistical analysis. The composite score integrated clinical findings, vaginal pH status, and fresh wet-mount microscopic findings recorded at each study visit.

For each diagnostic category, the composite score represented the sum of all predefined clinical, physiological, and microscopic variables: clinical findings contributed 1 point when present and 0 points when absent; vaginal pH contributed 1 point when classified as altered and 0 points when normal; and microscopic findings were graded from 0 to 3 points according to their abundance during fresh wet-mount examination. Component weighting was established *a priori* by the participating gynecologists to provide a standardized longitudinal summary of routinely collected clinical and microscopic variables and was not derived from statistical modeling or previous validation studies.

Because the characteristic manifestations of vulvovaginal candidiasis, bacterial vaginosis, and mixed vaginal infection differ, separate scoring frameworks were defined for each diagnostic category. The maximum possible score was 10 points for vulvovaginal candidiasis, 10 points for bacterial vaginosis, and 20 points for mixed vaginal infection.

Higher scores indicated a greater accumulation of predefined clinical, physiological, and microscopic abnormalities, whereas progressive reductions across follow-up visits reflected improvement in the variables included in the composite assessment.

The composite score was designed exclusively as an exploratory multidimensional longitudinal assessment framework to integrate routinely collected clinical manifestations, vaginal physiological changes, and microscopic findings into a single outcome measure. It was not intended to replace established diagnostic systems, including the Amsel criteria, Nugent score, or microbiological classification methods, nor should it be interpreted as a validated measure of disease severity or treatment response.

Disease severity categories and longitudinal response thresholds were predefined before statistical analysis. Based on the magnitude of score reduction between visits, treatment response was classified as no clinically meaningful improvement, minimal improvement, moderate improvement, or major improvement. The complete scoring framework and predefined response categories are summarized in Table 3.

### 2.7. Exploratory Longitudinal Score-Based Response Classification

To facilitate interpretation of treatment-associated changes over time, predefined response categories were established *a priori* before statistical analysis according to the magnitude of composite score reduction observed between study visits.

Because mixed vaginal infection included a broader range of clinical and microscopic variables and therefore had a higher maximum composite score than vulvovaginal candidiasis or bacterial vaginosis, disease-specific response thresholds were defined separately for each diagnostic category.

Response thresholds were based on proportional reductions in the disease-specific composite scores and were used exclusively to provide an exploratory characterization of longitudinal treatment-associated improvement. These response categories were not intended as validated responder definitions or clinical outcome measures.

The predefined response thresholds are summarized in Table 4.

### 2.8. Treatment

All included patients received intravaginal dequalinium chloride vaginal tablets (10 mg; Fluomizin^®^, Medinova AG, Zürich, Switzerland), commercially distributed in Mexico by Armstrong Laboratorios de México (Drug Registration No. 050M2021 SSA). Treatment consisted of one vaginal tablet administered once daily at bedtime for six consecutive days according to the manufacturer’s prescribing information and routine outpatient gynecological practice.

Treatment had been prescribed by the treating gynecologist during routine clinical care before initiation of this retrospective study. No study-specific therapeutic interventions were introduced for research purposes, and all treatment decisions were made independently of the study protocol.

To reduce potential confounding, only treatment episodes managed exclusively with intravaginal dequalinium chloride were eligible for inclusion. Patients who received concomitant antimicrobial, antifungal, probiotic, corticosteroid, or other therapies that could potentially influence assessment of treatment-associated outcomes were excluded.

### 2.9. Adverse Event Assessment

Safety evaluation was based on retrospective review of adverse events documented by the treating gynecologists during routine outpatient follow-up. No study-specific safety assessments were performed, and all safety data analyzed in the present study were obtained from medical records generated during routine clinical care before initiation of this retrospective investigation.

Any unfavorable medical occurrence temporally associated with intravaginal dequalinium chloride treatment and documented during treatment or follow-up visits was considered for safety evaluation. Reported adverse events were identified from the medical records and classified according to the Medical Dictionary for Regulatory Activities (MedDRA). Causality was assessed using the Naranjo Adverse Drug Reaction Probability Scale.

For each reported adverse event, available information regarding severity, duration, temporal relationship to treatment, clinical course, and outcome was extracted from the medical records. Safety results were summarized descriptively according to event frequency, MedDRA classification, and causality assessment.

### 2.10. Data Collection

Clinical data were retrospectively extracted from eligible medical records using a standardized data collection form developed before database construction. Data extraction was performed according to predefined protocol definitions to ensure consistency across participating investigators and reduce retrospective interpretative variability.

Collected variables included demographic characteristics, diagnostic classification, clinical findings, vaginal pH measurements, fresh wet-mount microscopic findings, disease-specific composite severity scores, longitudinal therapeutic response categories, treatment information, and safety outcomes.

Following extraction, all variables underwent quality review and verification according to predefined protocol criteria before inclusion in the final analytical database.

### 2.11. Outcomes

Primary Outcome

The primary outcome was the change in the disease-specific composite clinical–microscopic severity score between baseline evaluation (Visit 1) and post-treatment follow-up (Visit 3; Day 14).

Secondary Outcomes

Secondary outcomes included longitudinal changes in individual clinical manifestations, vaginal pH, fresh microscopic findings, restoration of lactobacillary predominance, disease severity categories, therapeutic response classifications, and safety outcomes.

Exploratory Outcomes

Exploratory analyses evaluated treatment-associated response patterns according to diagnostic classification (bacterial vaginosis, vulvovaginal candidiasis, and mixed vaginal infection) and characterized longitudinal relationships among clinical, physiological, and microscopic variables throughout follow-up.

### 2.12. Statistical Analysis

Statistical analyses were performed to evaluate longitudinal changes in clinical manifestations, vaginal pH, fresh wet-mount microscopic findings, and exploratory composite clinical–microscopic scores across the three predefined study visits.

Baseline continuous variables are summarized as mean ± standard deviation (SD), whereas longitudinal outcome variables are summarized as median and interquartile range (IQR). Categorical variables are summarized as frequencies and percentages.

Longitudinal comparisons among baseline (Visit 1), treatment completion (Visit 2; Day 7), and post-treatment follow-up (Visit 3; Day 14) were performed using the Friedman test for repeated measures. When statistically significant differences were identified, post hoc pairwise comparisons were performed using the Wilcoxon signed-rank test.

Prespecified subgroup analyses were conducted according to diagnostic classification, including bacterial vaginosis, vulvovaginal candidiasis, and mixed vaginal infection. Safety outcomes were analyzed descriptively according to MedDRA classification and Naranjo causality assessment.

All statistical analyses were two-sided, and a *p*-value < 0.05 was considered statistically significant.

### 2.13. Ethical Considerations

The study was conducted in accordance with the ethical principles of the Declaration of Helsinki, applicable national regulations governing clinical research, and institutional policies for the protection of confidential health information. The study protocol was reviewed and approved by the corresponding Research and Ethics Committees under approval number CEI-004-20231128.

Because of the retrospective observational design, no experimental intervention, modification of treatment, or alteration of routine clinical management was performed. All diagnostic evaluations, therapeutic decisions, microscopic examinations, and follow-up assessments corresponded exclusively to standard gynecological care provided by the treating physicians.

All data were retrospectively extracted from medical records and anonymized before database construction and statistical analysis. Personally identifiable information was removed from analytical datasets, and access to study data was restricted to authorized investigators in accordance with institutional confidentiality policies and applicable data protection regulations.

## 3. Results

### 3.1. Baseline Clinical, Physiological, and Microscopic Characteristics of the Study Population

A total of 69 non-pregnant women with complete longitudinal clinical and follow-up information were included in the final analytical cohort and classified as having vulvovaginal candidiasis (n = 24), bacterial vaginosis (n = 31), or mixed vaginal infection (n = 14). Patient selection, eligibility assessment, and diagnostic classification are summarized in Figure 1, and baseline demographic, anthropometric, reproductive, and disease-related characteristics are presented in Table 5.

The study population demonstrated demographic and reproductive characteristics consistent with those commonly observed among women of reproductive age affected by vulvovaginal infectious disorders. Age, anthropometric measures, age at menarche, and age at sexual debut were generally comparable across diagnostic groups.

The mean number of sexual partners during the preceding year remained low throughout the cohort. However, women with mixed vaginal infection reported the highest lifetime number of sexual partners (7.29 ± 7.37), compared with patients with bacterial vaginosis (4.13 ± 2.47) and vulvovaginal candidiasis (3.42 ± 2.10). Previous vaginal infections during the preceding year were documented in 37.7% of the study population, suggesting recurrent susceptibility in a substantial proportion of patients.

Baseline physiological assessment demonstrated higher vaginal pH values among women with bacterial vaginosis (6.71 ± 0.86) and mixed vaginal infection (6.71 ± 1.20) compared with vulvovaginal candidiasis (5.33 ± 1.13), consistent with the expected pathophysiological characteristics of these infectious phenotypes.

Overall, no major differences in demographic or reproductive characteristics were observed among diagnostic categories, supporting subsequent longitudinal comparisons of clinical, physiological, and microscopic outcomes.

### 3.2. Baseline Clinical, Physiological, and Microscopic Findings

Baseline clinical, physiological, and fresh microscopic findings are summarized descriptively according to diagnostic classification in Table 6. These variables were recorded at the initial visit before dequalinium chloride treatment and are presented as median (IQR) or frequency and percentage, as appropriate.

Among patients with vulvovaginal candidiasis, the most frequent baseline findings were thick white discharge (100%), vulvovaginal pruritus (95.8%), and yeasts on fresh microscopy (95.8%). Genital burning or irritation was documented in 54.2% of cases, while fetid discharge, homogeneous discharge, clue cells, reduced or absent lactobacilli, and pseudohyphae were not observed in this subgroup.

Among patients with bacterial vaginosis, the most frequent baseline findings were positive amine test (93.5%), fetid discharge (87.1%), clue cells (80.6%), reduced or absent lactobacilli (77.4%), and homogeneous discharge (74.2%). Vulvovaginal pruritus was documented in 3.2% of cases, whereas genital burning or irritation, thick white discharge, yeasts, and pseudohyphae were not observed.

Among patients with mixed vaginal infection, baseline findings included vulvovaginal pruritus (100%), clue cells (100%), yeasts on microscopy (85.7%), genital burning or irritation (85.7%), reduced or absent lactobacilli (71.4%), fetid discharge (64.3%), positive amine test (57.1%), and thick white discharge (42.9%). Pseudohyphae or pseudomycelia were identified in one patient (7.1%).

### 3.3. Overall Longitudinal Clinical–Microscopic Response

**Figure 2:**
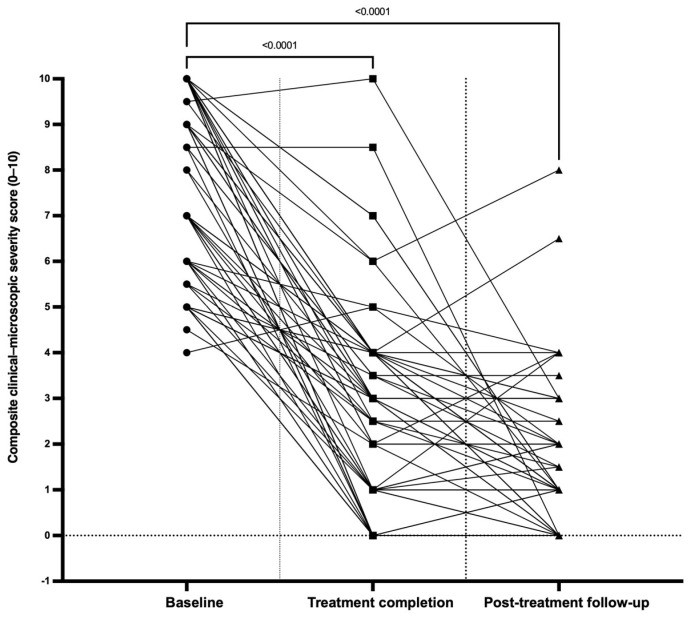
Global longitudinal reduction in composite clinical–microscopic severity score following dequalinium chloride therapy. Individual paired trajectories of the sixty-nine research subjects are shown for the overall cohort across baseline, treatment completion, and post-treatment follow-up. The composite clinical–microscopic severity score was normalized to a unified 0–10 scale before pooling diagnostic groups to avoid distortion from the higher raw scoring range used in mixed vaginal infection. Central tendency is presented as the median with the interquartile range. A longitudinal comparison was performed using a Friedman test.

The overall study population demonstrated progressive improvement across all evaluated domains following intravaginal dequalinium chloride therapy (Figure 2 and Table 7). Longitudinal reductions were observed in the number of clinical symptoms, vaginal pH values, clinical discomfort scores, fresh microscopic scores, and composite clinical–microscopic severity scores from baseline to treatment completion, with further improvement in or maintenance of response during post-treatment follow-up.

At baseline, the median number of symptoms was three (IQR 3–4), decreasing to one (IQR 0–1) at treatment completion and zero (IQR 0–1) at follow-up (*p* < 0.0001). Median vaginal pH decreased from 6 (IQR 5–7) at baseline to 5 (IQR 4–5) at both subsequent evaluations (*p* < 0.0001). Similar longitudinal reductions were observed for clinical discomfort, fresh microscopic, and composite clinical–microscopic severity scores (Table 7), with all comparisons reaching statistical significance (*p* < 0.0001).

### 3.4. Longitudinal Response According to Diagnostic Classification

**Figure 3:**
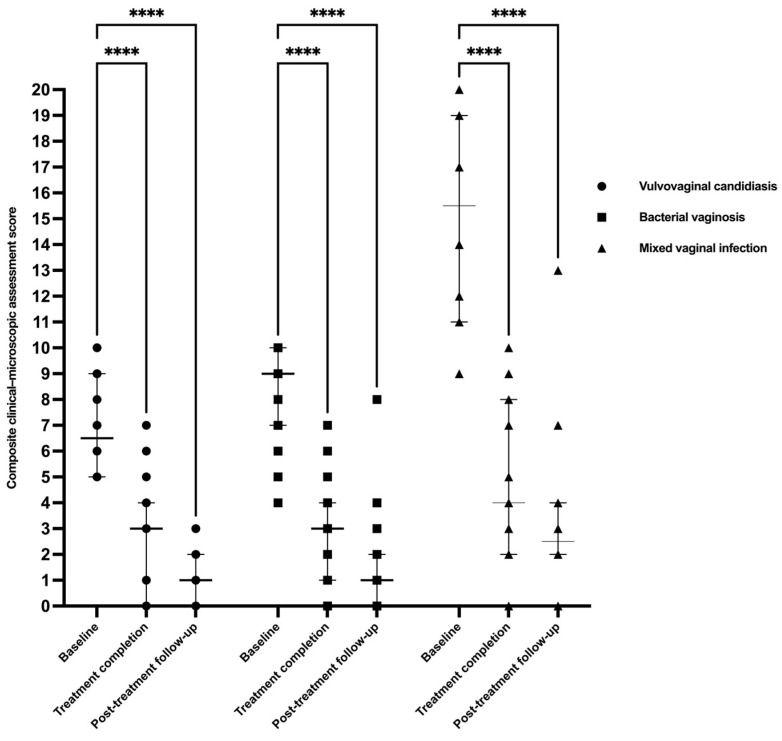
Longitudinal changes in composite clinical–microscopic scores within each diagnostic category. Data are presented as medians ± 95% confidence intervals. Overall longitudinal differences were analyzed using the Friedman test, followed by Wilcoxon signed-rank tests for pairwise comparisons. **** *p* < 0.0001 versus baseline.

Longitudinal improvement was observed within each diagnostic category following intravaginal dequalinium chloride therapy (Figure 3 and Table 8 and Table 9). Median composite clinical–microscopic scores progressively decreased from baseline to treatment completion and remained lower at post-treatment follow-up in patients with vulvovaginal candidiasis, bacterial vaginosis, and mixed vaginal infection.

Among patients with vulvovaginal candidiasis, the median number of symptoms decreased from three (IQR 2–3) at baseline to zero (IQR 0–1) at treatment completion and remained at zero (IQR 0–0) during follow-up (*p* < 0.0001). Median vaginal pH decreased from 5 to 4, while the composite clinical–microscopic severity score decreased from 6.5 (IQR 5–9) to 1 (IQR 0–2.25) at follow-up (*p* < 0.0001).

Patients with bacterial vaginosis demonstrated similar improvement: the median number of symptoms decreased from three (IQR 3–4) to zero (IQR 0–1), vaginal pH decreased from 7 to 5, and the composite clinical–microscopic severity score decreased from nine (IQR 7–10) to one (IQR 0–3) at follow-up (all *p* < 0.0001).

Among patients with mixed vaginal infection, symptom burden decreased from four (IQR 4–5.75) to one (IQR 0–1.75), vaginal pH decreased from 7 to 5, and the composite clinical–microscopic severity score decreased from 15.5 (IQR 11.25–17) to 4 (IQR 3–5) during follow-up (all *p* ≤ 0.0001). Although fresh microscopic scores improved markedly after treatment completion, residual microscopic findings persisted during post-treatment evaluation, resulting in a slower microscopic recovery profile compared with the other diagnostic groups. Nevertheless, substantial reductions were observed across all evaluated domains, including symptom burden, clinical discomfort, vaginal pH, and overall composite severity scores.

The exploratory score-derived response classification demonstrated progressive improvement across all diagnostic groups (Table 9). According to the predefined score-based response categories, 95.8%, 96.8%, and 92.9% of patients with vulvovaginal candidiasis, bacterial vaginosis, and mixed vaginal infection met the predefined responder criteria at treatment completion, respectively. At the post-treatment follow-up visit, all patients met at least the predefined minimal score-based improvement category, resulting in exploratory responder rates of 100% across the three diagnostic groups.

Overall, all longitudinal comparisons reached statistical significance, and the proportion of patients meeting the predefined exploratory responder categories increased throughout follow-up. These findings should be interpreted as score-derived longitudinal classifications rather than validated clinical response or cure rates. Although patients with mixed vaginal infection presented the highest baseline composite scores, all met at least the predefined minimal score-based improvement category at the final evaluation.

### 3.5. Safety and Tolerability

Intravaginal dequalinium chloride was generally well tolerated throughout follow-up (Table 10). All patients completed the prescribed treatment regimen, and no treatment discontinuations were documented.

A total of 13 adverse events were reported in 11 patients (15.9%) during follow-up evaluation. The most frequently reported event was mild lower abdominal or pelvic discomfort, affecting seven patients and accounting for nine recorded events, and transient vulvovaginal irritation and local intolerance manifestations (discomfort, dryness and/or burning sensation) were each reported in two patients.

All adverse events were classified as mild and self-limited, and resolved without clinically significant sequelae. No severe adverse events, treatment-related hospitalizations, serious safety concerns, or clinically significant deterioration was observed during follow-up.

Taken together, these findings indicate that intravaginal dequalinium chloride was associated with significant clinical, physiological, and microscopic improvement across all diagnostic categories while maintaining a favorable tolerability profile throughout follow-up.

## 4. Discussion

The present real-world longitudinal study provides additional evidence on the use of intravaginal dequalinium chloride by demonstrating consistent improvement in clinical manifestations, vaginal physiological parameters, and fresh wet-mount microscopic findings among women with vulvovaginal candidiasis, bacterial vaginosis, and mixed vaginal infection. Improvement became evident at treatment completion and was sustained throughout the short-term follow-up period. Although women with mixed vaginal infection presented the greatest baseline disease burden and a slower pattern of microscopic recovery, clinically meaningful improvement was observed across all diagnostic groups by the end of follow-up. Collectively, these observations suggest that intravaginal dequalinium chloride may represent a useful broad-spectrum local therapeutic option for heterogeneous vulvovaginal infectious disorders encountered in routine gynecological practice, while recognizing the inherent limitations of a retrospective observational study.

The findings of the present study are consistent with previous investigations supporting the efficacy of intravaginal dequalinium chloride in the management of vulvovaginal infectious disorders. A multicenter randomized clinical trial demonstrated that dequalinium chloride was noninferior to intravaginal clindamycin, achieving comparable clinical cure rates while maintaining a favorable safety profile [12]. Similarly, more recent evidence showed noninferiority to oral metronidazole for the treatment of bacterial vaginosis, with comparable therapeutic outcomes and favorable tolerability [10]. Reviews and expert assessments have further concluded that dequalinium chloride represents an effective locally administered therapeutic option because of its broad antimicrobial activity, favorable tolerability profile, and low potential for resistance development [14,17,18]. The present study builds upon these observations by demonstrating that therapeutic improvement was not limited to symptom resolution alone but was accompanied by concordant normalization of vaginal physiological parameters and fresh microscopic findings. This multidimensional response pattern may provide a more comprehensive representation of treatment-associated recovery under routine clinical practice conditions than clinical outcomes alone.

The favorable outcomes observed in the present study may be related to the broad-spectrum antiseptic activity of dequalinium chloride. Unlike conventional antibiotics that target specific bacterial metabolic pathways, dequalinium chloride acts through multiple mechanisms involving disruption of microbial cell membrane integrity and cellular permeability, resulting in activity against Gram-positive and -negative bacteria, anaerobic microorganisms, fungi, and protozoa commonly implicated in vulvovaginal infections [17]. Experimental studies have confirmed activity against a wide range of vaginal pathogens, supporting its use across diverse infectious presentations [19]. The broad antimicrobial spectrum of dequalinium chloride may partially explain the concordant clinical, physiological, and microscopic improvements observed across bacterial vaginosis, vulvovaginal candidiasis, and mixed vaginal infection in the present cohort.

Despite the growing body of evidence supporting intravaginal dequalinium chloride, important knowledge gaps remain. Most published studies have been conducted in European populations and have primarily focused on clinical cure or symptom resolution as the principal therapeutic endpoints. In routine gynecological practice, however, treatment response is commonly assessed through the combined interpretation of clinical manifestations, vaginal pH, and fresh wet-mount microscopic findings. The present study addresses this gap by providing real-world longitudinal evidence from a Mexican outpatient population and by integrating serial assessment of clinical manifestations, vaginal pH, fresh wet-mount microscopy, and exploratory composite clinical–microscopic severity scores across women with vulvovaginal candidiasis, bacterial vaginosis, and mixed vaginal infection. This multidimensional approach allowed for a more comprehensive characterization of treatment-associated recovery than symptom-based evaluation alone and provides complementary real-world evidence to the existing clinical trial literature.

In the present study, favorable longitudinal evolution was observed among women with mixed vaginal infection, which represents a clinically challenging condition because it combines characteristics of bacterial dysbiosis and fungal overgrowth. In the present cohort, patients with mixed vaginal infection exhibited the highest baseline composite clinical–microscopic severity scores together with the greatest microscopic burden among all evaluated infectious profiles. These findings are consistent with the coexistence of bacterial and fungal alterations simultaneously affecting the vaginal microenvironment [20]. Despite this greater baseline disease burden and a slower pattern of microscopic recovery, clinically meaningful changes were achieved in all patients by the end of follow-up, suggesting that dequalinium chloride may remain effective even in more complex vulvovaginal infectious presentations characterized by overlapping microbiological and clinical abnormalities.

Current evidence indicates that bacterial vaginosis is frequently associated with polymicrobial communities and biofilm formation, factors that contribute to persistence and recurrence despite initial clinical improvement [21,22,23]. In the present study, longitudinal assessment was based on vaginal pH and fresh wet-mount microscopy rather than standardized microbiological reference methods such as Nugent scoring, quantitative assessment of Lactobacillus spp., microbiological culture, molecular diagnostic techniques, or microbiome sequencing. Consequently, the observed reductions in clue cells, inflammatory findings, and alterations of the vaginal flora, together with progressive normalization of vaginal pH, should be interpreted as evidence of improvement in routinely assessed clinical and microscopic parameters rather than as direct evidence of restoration of the vaginal microbiome. Future prospective studies incorporating standardized microbiological techniques and longer follow-up are needed to determine whether these early changes are associated with sustained clinical remission and reduced recurrence.

Although biofilm formation and recurrence were not specifically evaluated in the present study, the observed reductions in clue cells, inflammatory findings, and alterations in vaginal flora, together with progressive normalization of vaginal pH, are consistent with improvement in routinely assessed clinical and microscopic parameters. However, because longitudinal evaluation relied on vaginal pH assessment and fresh wet-mount microscopy rather than standardized microbiological reference methods, these findings should not be interpreted as direct evidence of restoration of the vaginal microbiome. Future studies incorporating Nugent scoring, quantitative assessment of Lactobacillus spp., molecular diagnostic techniques, or microbiome sequencing are needed to determine whether these early changes are associated with sustained microbiological recovery and reduced recurrence.

Interestingly, median vaginal pH in the vulvovaginal candidiasis subgroup was slightly higher than typically reported for uncomplicated candidiasis, as diagnostic classification was based on the concordance of clinical presentation, fresh microscopy, and available microbiological confirmation rather than vaginal pH alone.

From a practical clinical perspective, these findings suggest that assessment of treatment response in vulvovaginal infectious disorders may benefit from incorporating physiological and microscopic parameters in addition to symptom evaluation alone. The concordant improvement observed across clinical manifestations, vaginal pH, and fresh microscopic findings supports the value of a multidimensional approach for monitoring short-term treatment response during routine gynecological practice. However, the present study was limited to short-term follow-up and therefore cannot determine whether these early improvements translate into lower recurrence rates or sustained clinical remission. Prospective studies with longer follow-up are required to address these outcomes.

The findings should be interpreted within the context of the retrospective, uncontrolled design of the study. Because all patients received intravaginal dequalinium chloride and no comparator group was available, the observed improvements cannot be attributed exclusively to treatment. As with any single-arm observational study, spontaneous symptom fluctuation, regression to the mean, diagnostic misclassification, behavioral changes, placebo responses, unmeasured confounding, and other sources of bias may have contributed to the observed longitudinal evolution. In addition, only 69 of the 230 screened medical records fulfilled all predefined eligibility criteria, with the principal reason for exclusion being the absence of complete baseline fresh microscopic documentation required for standardized longitudinal assessment. Although diagnostic classification had been established previously by the treating gynecologist during routine clinical care, incomplete baseline microscopic documentation precluded standardized longitudinal evaluation, potentially limiting the representativeness of the final analytical cohort. Accordingly, these findings should be interpreted as descriptive, hypothesis-generating real-world evidence rather than evidence of comparative efficacy or causality.

Treatment tolerability was assessed retrospectively from adverse events documented during routine clinical care rather than through standardized prospective safety assessments or active surveillance. Consequently, mild or transient adverse events may have been underreported if they were not documented in the medical records. Nevertheless, the favorable tolerability profile observed in the present cohort is consistent with the safety findings reported in randomized clinical trials and recent systematic reviews evaluating intravaginal dequalinium chloride for vulvovaginal infections [11,14].

## 5. Conclusions

In this retrospective real-world study, intravaginal dequalinium chloride treatment was associated with short-term improvement in clinical manifestations, vaginal pH, fresh wet-mount microscopic findings, and exploratory composite clinical–microscopic scores among women with vulvovaginal candidiasis, bacterial vaginosis, and mixed vaginal infection. The concordant improvement observed across clinical, physiological, and microscopic parameters supports the potential value of a multidimensional approach for evaluating treatment response during routine gynecological practice. Because of the retrospective uncontrolled design and the absence of standardized microbiological reference methods, these findings should be interpreted as descriptive, hypothesis-generating real-world evidence rather than evidence of comparative efficacy, microbiological cure, or causality. Prospective controlled studies incorporating standardized microbiological endpoints and longer follow-up are warranted to confirm these findings and to evaluate recurrence, long-term safety, and the durability of treatment response.

## Figures and Tables

**Table 1:** Clinical variables incorporated into the disease-specific severity assessment framework. Clinical variables included in the disease-specific clinical assessment framework. The presence or absence of a variable contributed 1 or 0 points, respectively, to the clinical assessment score.

Clinical Variable	Vulvovaginal Candidiasis	Bacterial Vaginosis	Mixed Vaginal Infection
Vaginal Discharge	1	1	1
Fetid Discharge	0	1	1
Homogeneous Discharge	0	1	1
Vulvovaginal Pruritus	1	0	1
Burning/Irritation	1	0	1
Positive Amine Whiff Test (KOH Test)	0	1	1

**Table 2:** Semi-quantitative grading system for fresh wet-mount microscopic findings. A semi-quantitative microscopic grading system used for longitudinal assessment. Microscopic findings were scored from 0 to 3 points according to their abundance during fresh wet-mount examination, with higher scores indicating greater microbiological abnormality.

Microscopic Finding	0 Points	1 Point	2 Points	3 Points
Clue cells	Absent	Scarce	Moderate	Abundant
Leukocytes/ neutrophils	Absent or 1–2 per field	Mild increase	Moderate increase	Marked increase
Bacillary flora alteration	Normal	Moderate alteration	Scarce bacilli	Absent bacilli
Yeasts	Absent	Scarce	Moderate	Abundant
Pseudohyphae	Absent	Scarce	Moderate	Abundant
Additional abnormal findings	Absent	Scarce	Moderate	Abundant

**Table 3:** Disease-specific exploratory composite assessment framework. Composite scores were calculated by summing disease-specific clinical variables (0–1 point each), vaginal pH status (0–1 point), and semi-quantitative microscopic findings (0–3 points each). Separate scoring frameworks were established for vulvovaginal candidiasis, bacterial vaginosis, and mixed vaginal infection according to their characteristic clinical and microbiological features. Higher scores indicate a greater accumulation of predefined clinical, physiological, and microscopic abnormalities. Categories were established *a priori* exclusively for exploratory longitudinal assessment and should not be interpreted as validated disease severity classifications. Severity categories were defined *a priori* according to proportional distribution across the maximum achievable score for each diagnostic category and were intended exclusively for exploratory longitudinal assessment rather than diagnostic classification or validation purposes.

Parameter	Vulvovaginal Candidiasis	Bacterial Vaginosis	Mixed Vaginal Infection
Maximum total score	10	10	20
Lowest predefined score range	<3	<3	<6
Low predefined score range	3–5	3–5	6–10
Intermediate predefined score range	6–8	6–8	11–16
High predefined score range	9–10	9–10	17–20

**Table 4:** Longitudinal therapeutic response classification. Disease-specific thresholds used to categorize the magnitude of treatment-associated improvement according to changes in composite severity scores between study visits. Response categories were defined before statistical analysis and classified as no clinically meaningful improvement, minimal improvement, moderate improvement, or major improvement. Larger reductions in composite scores were interpreted as greater resolution of symptoms, normalization of vaginal physiology, and improvement of microscopic findings.

Score Reduction Between Visits	Vaginal Candidiasis/ Bacterial Vaginosis	Mixed Vaginal Infection	Clinical Interpretation
No predefined score-based improvement	0–1.9 points	0–3.9 points	No improvement
Minimal reduction	2–4.9 points	4–9.9 points	Minimal improvement
Moderate reduction	5–7.9 points	10–15.9 points	Moderate improvement
Major reduction	8–10 points	16–20 points	Major improvement

**Table 5:** Baseline demographic, anthropometric, and reproductive characteristics according to diagnostic classification. Baseline demographic, anthropometric, and reproductive characteristics stratified according to diagnostic classification. Continuous variables are reported as mean ± standard deviation (SD), whereas categorical variables are presented as frequencies and percentages.

Variable	Vulvovaginal Candidiasis (n = 24)	Bacterial Vaginosis (n = 31)	Mixed Vaginal Infection (n = 14)	Total Population (n = 69)
Demographic and anthropometric variables				
Age (years), mean ± SD	34.67 ± 9.72	32.00 ± 7.44	35.43 ± 5.65	33.62 ± 8.04
Weight (kg), mean ± SD	64.37 ± 12.43	61.14 ± 8.44	64.86 ± 9.66	63.01 ± 10.22
Height (cm), mean ± SD	161.63 ± 4.00	160.45 ± 3.87	159.86 ± 6.57	160.74 ± 4.55
Reproductive characteristics				
Menarche age (years), mean ± SD	12.08 ± 1.74	12.13 ± 1.57	12.43 ± 1.70	12.17 ± 1.63
Age at onset of sexual activity (years), mean ± SD	18.46 ± 3.58	18.55 ± 3.06	18.21 ± 2.83	18.44 ± 3.16
Sexual partners during previous year, mean ± SD	1.08 ± 0.28	1.23 ± 0.67	1.86 ± 1.41	1.30 ± 0.82
Lifetime number of sexual partners, mean ± SD	3.42 ± 2.10	4.13 ± 2.47	7.29 ± 7.37	4.52 ± 4.08
Previous vaginal infection during preceding year, n (%)	8 (33.3%)	11 (35.5%)	7 (50.0%)	26 (37.7%)

**Table 6:** Baseline clinical, physiological, and fresh microscopic characteristics according to diagnostic classification. Data are presented as median (interquartile range [IQR]) or frequency and percentage, as appropriate. All findings correspond to the baseline evaluation before initiation of intravaginal dequalinium chloride therapy. The composite severity score corresponds to the disease-specific clinical–microscopic scoring framework described in the Methods Section. Reduced or absent lactobacilli refers to decreased Döderlein bacillary predominance identified during fresh wet-mount examination. A positive amine (“whiff”) test corresponds to abnormal odor release after potassium hydroxide application during gynecological assessment.

Variable	Vulvovaginal Candidiasis (n = 24)	Bacterial Vaginosis (n = 31)	Mixed Infection (n = 14)	Total (n = 69)
Vaginal pH, median (IQR)	5.0 (5–6)	7.0 (6–7)	7.0 (6–7)	6.0 (5–7)
Vulvovaginal pruritus, n (%)	23 (95.8%)	1 (3.2%)	14 (100%)	38 (55.1%)
Genital burning/irritation, n (%)	13 (54.2%)	0 (0%)	12 (85.7%)	25 (36.2%)
Thick white discharge, n (%)	24 (100%)	0 (0%)	6 (42.9%)	30 (43.5%)
Fetid discharge, n (%)	0 (0%)	27 (87.1%)	9 (64.3%)	36 (52.2%)
Homogeneous discharge, n (%)	0 (0%)	23 (74.2%)	5 (35.7%)	28 (40.6%)
Positive amine test, n (%)	1 (4.2%)	29 (93.5%)	8 (57.1%)	38 (55.1%)
Yeasts on microscopy, n (%)	23 (95.8%)	0 (0%)	12 (85.7%)	35 (50.7%)
Clue cells, n (%)	0 (0%)	25 (80.6%)	14 (100%)	39 (56.5%)
Reduced/absent lactobacilli, n (%)	0 (0%)	24 (77.4%)	10 (71.4%)	34 (49.3%)
Pseudohyphae/pseudomycelia, n (%)	0 (0%)	0 (0%)	1 (7.1%)	1 (1.4%)

**Table 7:** Overall longitudinal treatment response, physiological, and fresh microscopic evolution following intravaginal dequalinium chloride therapy. Longitudinal evolution of clinical, physiological, microscopic, and composite severity assessment variables following intravaginal dequalinium chloride therapy. Data are presented as median (IQR). Overall longitudinal comparisons across study visits were performed using the Friedman test. Global and multiple comparisons between the three groups showed a *p* value < 0.0001. For pooled analyses, composite severity scores were standardized to a common 0–10 scale before combining diagnostic groups because mixed vaginal infection used a higher raw scoring range than vulvovaginal candidiasis and bacterial vaginosis. Disease-specific analyses were performed using the original raw scores.

Overall Cohort (All Diagnostic Groups Combined)	Baseline (Pre-CDQ) Median (IQR)	Treatment Completion Median (IQR)	Post-Treatment Follow-Up Median (IQR)	*p*-Value (Friedman)
Number of symptoms	3 (3–4)	1 (0–1)	0 (0–1)	<0.0001
Vaginal pH	6 (5–7)	5 (4–5)	5 (4–5)	<0.0001
Clinical discomfort score	4 (3–4)	1 (0–1)	0 (0–1)	<0.0001
Fresh microscopy score	6 (3–6)	3 (0–3)	1.5 (0–3)	<0.0001
Composite clinical– microscopic severity score	8 (6–10)	3 (1–5)	1 (0–3)	<0.0001

**Table 8:** Longitudinal evolution of clinical, physiological, microscopic, and composite severity score outcomes according to diagnostic classification following dequalinium chloride therapy. Data are presented as median (interquartile range [IQR]). Longitudinal comparisons were performed using the Friedman test. Lower composite clinical–microscopic severity scores indicate lower disease severity.

Diagnostic Group	Parameter	Baseline Median (IQR)	Treatment Completion Median (IQR)	Post-Treatment Follow-Up Median (IQR)	*p*-Value (Friedman)
Vulvovaginal candidiasis	Number of symptoms	3 (2–3)	0 (0–1)	0 (0–0)	<0.0001
	Vaginal pH	5 (5–6)	4 (4–5)	4 (4–5)	<0.0001
	Clinical discomfort score	3 (2–3)	0 (0–1)	0 (0–0)	<0.0001
	Fresh microscopic score	3 (3–6)	3 (0–3)	0.5 (0–2.25)	<0.0001
	Composite clinical–microscopic score	6.5 (5–9)	3 (0–4)	1 (0–2.25)	<0.0001
Bacterial vaginosis	Number of symptoms	3 (3–4)	1 (0–1)	0 (0–1)	<0.0001
	Vaginal pH	7 (6–7)	5 (4–5)	5 (4–5)	<0.0001
	Clinical discomfort score	4 (3–4)	1 (0–1)	0 (0–1)	<0.0001
	Fresh microscopic score	6 (3–6)	3 (0–3)	0 (0–3)	<0.0001
	Composite clinical–microscopic score	9 (7–10)	3 (1–4)	1 (0–3)	<0.0001
Mixed vaginal infection	Number of symptoms	4 (4–5.75)	1 (1–2)	1 (0–1.75)	<0.0001
	Vaginal pH	7 (6–7)	5 (4.12–7)	5 (4–6)	0.0001
	Clinical discomfort score	5 (5–6.5)	2 (1–2)	1 (0–2)	<0.0001
	Fresh microscopic score	9 (6.75–12)	3 (3–6)	3 (3–3)	<0.0001
	Composite clinical–microscopic score	15.5 (11.25–17)	6 (5–7.75)	4 (3–5)	<0.0001

IQR, interquartile range.

**Table 9:** Distribution of exploratory therapeutic response categories according to diagnostic classification following dequalinium chloride therapy. Data are presented as frequency and percentage. Therapeutic response categories were defined according to predefined reductions in the composite clinical–microscopic severity score. For vulvovaginal candidiasis and bacterial vaginosis, no clinically meaningful improvement, minimal improvement, moderate improvement, and major improvement corresponded to score reductions of <2, 2–4.9, 5–7.9, and ≥8 points, respectively. For mixed vaginal infection, corresponding thresholds were <4, 4–9.9, 10–15.9, and ≥16 points, respectively. Responders were defined as patients achieving at least minimal clinically meaningful improvement.

Diagnostic Group	Comparison	Improvement				Responders n (%)
		No Changes n (%)	Minimal n (%)	Moderate n (%)	Major n (%)	
Vulvovaginal candidiasis	Baseline → Day 7	1 (4.2%)	5 (20.8%)	10 (41.7%)	8 (33.3%)	23 (95.8%)
	Baseline → Day 14	0 (0%)	3 (12.5%)	8 (33.3%)	13 (54.2%)	24 (100%)
Bacterial vaginosis	Baseline → Day 7	1 (3.2%)	6 (19.4%)	13 (41.9%)	11 (35.5%)	30 (96.8%)
	Baseline → Day 14	0 (0%)	4 (12.9%)	11 (35.5%)	16 (51.6%)	31 (100%)
Mixed Vaginal infection	Baseline → Day 7	1 (7.1%)	4 (28.6%)	6 (42.9%)	3 (21.4%)	13 (92.9%)
	Baseline → Day 14	0 (0%)	3 (21.4%)	5 (35.7%)	6 (42.9%)	14 (100%)
Overall cohort	Baseline → Day 7	3 (4.3%)	15 (21.7%)	29 (42.0%)	22 (31.9%)	66 (95.7%)
	Baseline → Day 14	0 (0%)	10 (14.5%)	24 (34.8%)	35 (50.7%)	69 (100%)

**Table 10:** Adverse events and tolerability findings during follow-up after dequalinium chloride therapy. Adverse events were identified during routine clinical follow-up and classified according to severity and outcome. All reported events were mild, self-limited, and resolved without treatment discontinuation or clinically significant sequelae. No serious adverse events or treatment-related hospitalizations were observed.

Adverse Event	Patients n (%)	Number of Events	Severity	Outcome
Mild lower abdominal/ pelvic discomfort	7 (10.1%)	9	Mild	Resolved without sequelae
Transient vulvovaginal irritation	2 (2.9%)	2	Mild	Resolved without sequelae
Local intolerance manifestations	2 (2.9%)	2	Mild	Resolved without sequelae
Treatment discontinuation	0 (0%)	0	Not observed	Not applicable
Serious adverse events	0 (0%)	0	Not observed	Not applicable
Hospitalization related to treatment	0 (0%)	0	Not observed	Not applicable

## Data Availability

The datasets generated and analyzed during the current study are available from the corresponding author upon reasonable request. Any shared dataset will be fully anonymized to protect patient confidentiality and will be provided in accordance with institutional and ethical regulations.

## Supplementary Materials

The following supporting information can be downloaded at https://www.mdpi.com/article/10.3390/jcm15156115/s1: Supplementary Figure S1: Representative fresh wet-mount microscopic findings in vulvovaginal candidiasis, bacterial vaginosis, and mixed vaginal infection.

## Abbreviations

The following abbreviations are used in this manuscript:

BV	bacterial vaginosis
CDQ	dequalinium chloride
IQR	interquartile range
KOH	potassium hydroxide
RCT	randomized controlled trial
SD	standard deviation
VVC	vulvovaginal candidiasis
