# Supplementary material for: Longitudinal Clinical and Microscopic Findings After Dequalinium Chloride Treatment in Vulvovaginal Infections: A Retrospective Real-World Study from Mexican Gynecological Practice
Source: J Clin Med. 2026 Aug 6;15(15):6115. doi: 10.3390/jcm15156115 (PMC13466461; doi:10.3390/jcm15156115)
Supplement: Supplementary file 1 [file jcm-15-06115-s001.zip › jcm-4405588-supplementary.pdf]

Figure S1. Representative fresh wet-mount microscopic findings in vulvovaginal candidiasis, bacterial vaginosis, and mixed vaginal infection.

(A) Vulvovaginal candidiasis showing yeast forms compatible with *Candida spp.*, preserved lactobacillary flora, and occasional leukocytes.

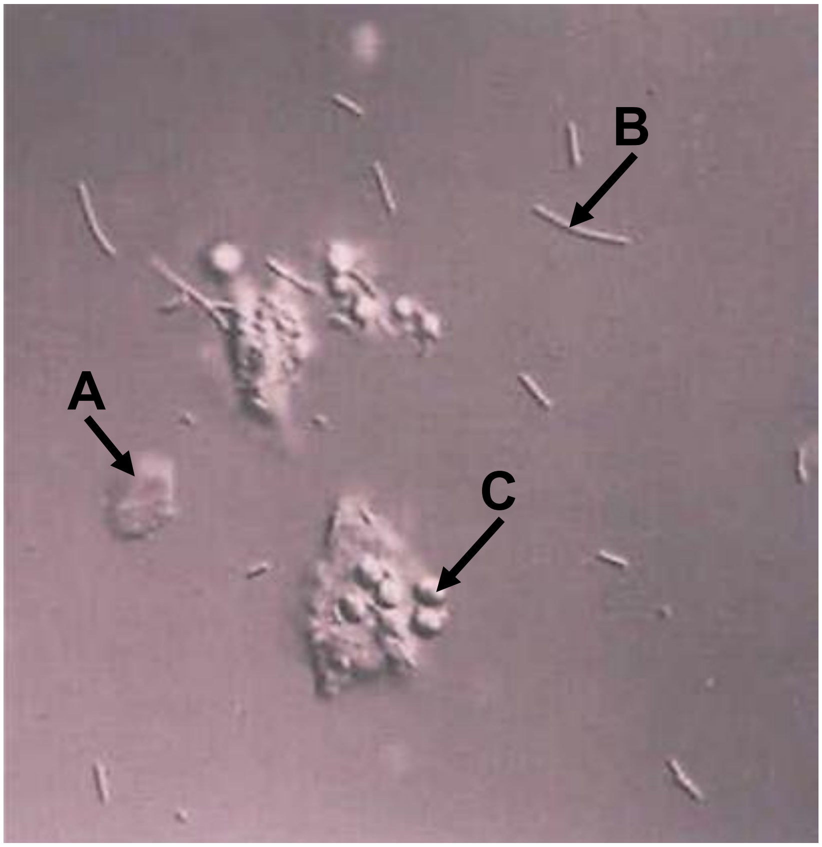

Figure S1A. Vulvovaginal candidiasis. Representative fresh wet-mount microscopy (bright-field microscopy, 40× objective) showing abundant yeast forms compatible with *Candida* spp. (>3 yeast cells per field). An isolated leukocyte is identified (A), abundant lactobacillary morphotypes consistent with preserved Döderlein flora are observed throughout the field (B), and multiple yeast cells attached to a vaginal epithelial cell are indicated (C).

(B) Bacterial vaginosis demonstrating a characteristic clue cell with extensive bacterial adherence and loss of normal cellular borders.

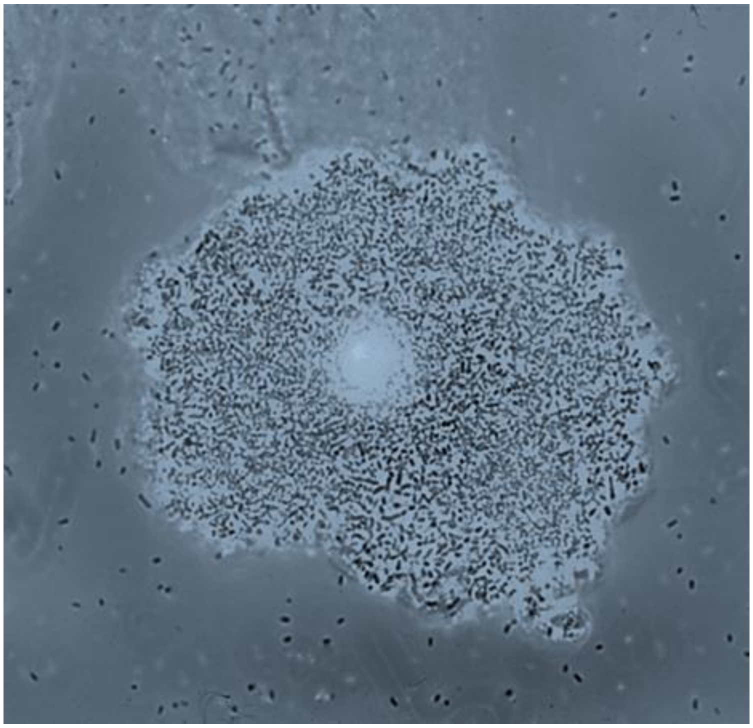

Figure S1B. Bacterial vaginosis. Representative fresh wet-mount microscopy (40× objective) demonstrating a characteristic clue cell with extensive bacterial adherence obscuring normal epithelial cell borders. The absence of surrounding lactobacillary morphotypes and the presence of dense bacterial colonization are findings consistent with bacterial vaginosis.

(C) Mixed vaginal infection showing simultaneous bacterial overgrowth and yeast structures compatible with concomitant bacterial vaginosis and vulvovaginal candidiasis.

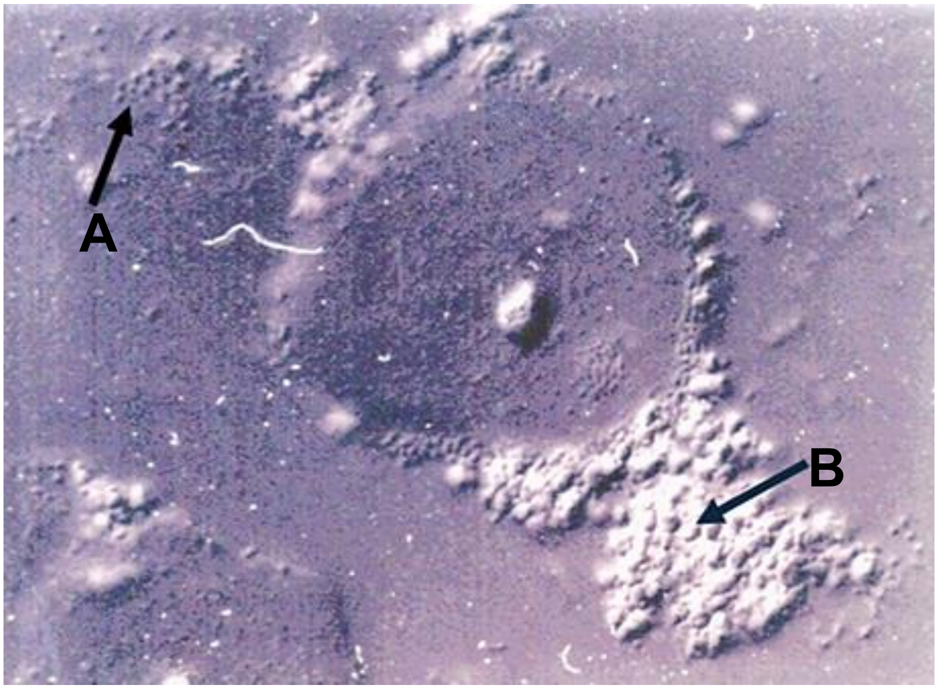

Figure S1C. Mixed vaginal infection. Representative fresh wet-mount microscopy (40× objective) demonstrating the coexistence of bacterial and fungal findings within the same specimen. A vaginal epithelial cell occupies the center of the field and is surrounded by numerous bacterial morphotypes (A), while a large aggregate of yeast forms compatible with *Candida* spp. is identified in the peripheral region (B). These concurrent microscopic findings are consistent with mixed vaginal infection involving bacterial vaginosis and vulvovaginal candidiasis.
